# Deciphering the role of nuclear and cytoplasmic IKKα in skin cancer

**DOI:** 10.18632/oncotarget.8792

**Published:** 2016-04-18

**Authors:** Josefa P. Alameda, Miriam Gaspar, Ángel Ramírez, Manuel Navarro, Angustias Page, Cristian Suárez-Cabrera, M. Guadalupe Fernández, Jose R. Mérida, Jesús M. Paramio, Rosa A. García-Fernández, M. Jesús Fernández-Aceñero, M. Llanos Casanova

**Affiliations:** ^1^ Molecular Oncology Unit, Centro de Investigaciones Energéticas, Medioambientales y Tecnológicas (CIEMAT), 28040 Madrid, Spain; ^2^ Molecular Oncology, Institute of Biomedical Investigation University Hospital “12 de Octubre”, 28041 Madrid, Spain; ^3^ Department of Human Anatomy and Embriology, Facultad de Medicina, UCM, 28040 Madrid, Spain; ^4^ Department of Animal Medicine and Surgery, Facultad de Veterinaria, UCM, 28040 Madrid, Spain; ^5^ Department of Pathology, Hospital Clínico San Carlos, 28040 Madrid, Spain

**Keywords:** nuclear IKKα, cytoplasmic IKKα, skin cancer, Maspin, c-Myc

## Abstract

Nonmelanoma skin cancers (NMSC) are the most common human malignancies. IKKα is an essential protein for skin development and is also involved in the genesis and progression of NMSC, through mechanisms not fully understood. While different studies show that IKKα protects against skin cancer, others indicate that it promotes NMSC. To resolve this controversy we have generated two models of transgenic mice expressing the IKKα protein in the nucleus (N-IKKα mice) or the cytoplasm (C-IKKα mice) of keratinocytes. Chemical skin carcinogenesis experiments show that tumors developed by both types of transgenic mice exhibit histological and molecular characteristics that make them more prone to progression and invasion than those developed by Control mice. However, the mechanisms through which IKKα promotes skin tumors are different depending on its subcellular localization; while IKKα of cytoplasmic localization increases EGFR, MMP-9 and VEGF-A activities in tumors, nuclear IKKα causes tumor progression through regulation of c-Myc, Maspin and Integrin-α6 expression. Additionally, we have found that N-IKKα skin tumors mimic the characteristics associated to aggressive human skin tumors with high risk to metastasize. Our results show that IKKα has different non-overlapping roles in the nucleus or cytoplasm of keratinocytes, and provide new targets for intervention in human NMSC progression.

## INTRODUCTION

The epidermis is a stratified squamous epithelium composed mainly of keratinocytes. Basal keratinocytes proliferate and give rise to differentiated cells, which, upon full maturation, generate the squamous cornified cell layer. Alterations in the normal physiology of the skin lead to numerous pathologies such as cancer. Keratinocyte derived non-melanoma skin cancer (NMSC) comprises two different entities: basal cell carcinoma (BCC) and cutaneous squamous cell carcinoma (SCC). NMSC is the most common form of cancer in the Caucasian population, representing 90% of skin cancers [1]. Nearly 5% of SCCs metastasize; therefore due to its high incidence, the mortality concomitant to aggressive cutaneous SCCs is reaching important numbers [2, 3].

IKKα is a member of the IKK complex, which is composed of two kinase subunits, IKKα and IKKβ, and a regulatory subunit, IKKγ/NEMO. While IKKβ is essential for NF-κB activation in the canonical pathway, regulating immunity and inflammation [4], IKKα is not required for these functions. The analysis of IKKα deficient mice showed that the main function of IKKα is the regulation of epidermal morphogenesis, as IKKα^−/−^ newborn mice exhibited a marked hyperplasic epidermis and lacked a terminally differentiated cornified layer [5–8]. Moreover, the kinase activity of IKKα activating the canonic pathway of NF-κB is not required for its function in skin development, as kinase-dead IKKα mutants are able to rescue the skin phenotype of IKKα^−/−^ mice [9]. IKKα plays, however, other relevant functions in the cytoplasm of cells, i.e., it is essential for signaling through the alternative NF-κB pathway, which activates the RelB/p52 heterodimer. This pathway is important for lymphoid organogenesis, B-cell survival and maturation, and adaptive immunity [10]. IKKα is also relevant for mammary epithelial proliferation signaling via cyclin D1 [11]. More recently it has been observed that IKKα translocates into the nucleus where, by targeting a growing list of substrates it acts on different biological functions including apoptosis, immune functions, cell proliferation, tumor suppression or progression, and chromatin remodeling [12–14]. For instance, it has been reported that in the cell nucleus IKKα regulates gene transcription: IKKα phosphorylates specific nuclear substrates such as histone H3, SMRT and N-CoR, thus regulating NF-κB dependent and independent transcription [12, 15, 16]. It also acts in the nucleus of epidermal cells as a cofactor for Smad2/3 in a Smad4-independent pathway that inhibits keratinocyte proliferation [17, 18].

There are numerous evidences indicating that IKKα functions in tumor progression in colorectal [15, 19], breast [20, 21], pancreatic [22], gastric [23], and prostatic [24, 25] cancers, as well as in hepatocarcinomas [26] and osteosarcomas [27]. Regarding the role that IKKα plays in the development and progression of NMSC, as mice expressing lower levels of IKKα develop more and larger skin tumors than control mice, it has been proposed that IKKα acts as a suppressor of skin carcinomas [28, 29]. However, we have found that IKKα has a pro-oncogenic role in skin cancer, as overexpression of IKKα in tumor epidermal cells (PDVC57) or in keratinocytes of transgenic mice increases the malignancy of cutaneous tumors [30, 31]; in addition, augmented levels of IKKα have been observed in some human cutaneous SCCs [31, 32]. In an attempt to explain this apparent controversy, it has been suggested that in skin tumors overexpressing IKKα most of it is in the cytoplasm, and that the loss of nuclear IKKα is likely the cause of the malignant conversion of keratinocytes [33–35]. Hence, to discern the relevance of the nuclear or cytoplasmic localization of IKKα for skin cancer development and progression, we have generated two new models of transgenic mice expressing human IKKα under the control of the bovine keratin *K5* promoter. By altering the nuclear localization signals, we have directed the exogenous IKKα protein towards the nucleus (N-IKKα mice) or the cytoplasm (C-IKKα mice) of keratinocytes in the basal, proliferative layer of the epidermis and in the outer root sheath of hair follicles. We here show that regardless of its subcellular localization, IKKα plays a protumoral role in skin cancer development and progression, although the mechanisms by which IKKα exerts its prooncogenic function are different depending on whether it acts in the nucleus or the cytoplasm of keratinocytes. Our results will help in understanding the progression of human NMSC; also offer new targets for intervention in such common cancer in humans. In addition, our C-and-N-IKKα transgenic mice provide an excellent model for dissecting the role of nuclear or cytoplasmic localization of IKKα in the physiology of the skin and other stratified epithelia.

## RESULTS

### Transgenic IKKα is expressed in the cytoplasm or in the nucleus of keratinocytes of C-and N-IKKα mice respectively

**Figure 1:**
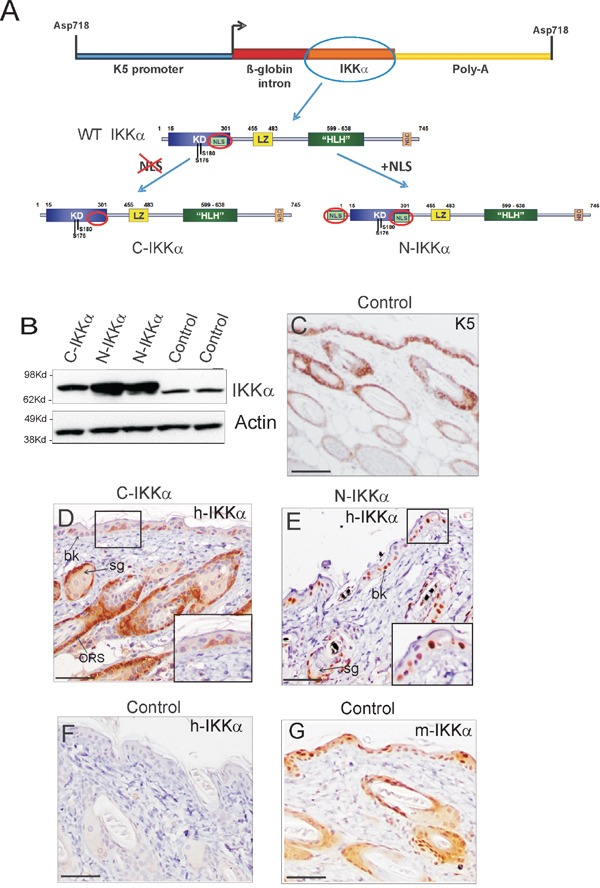
Expression of the transgenic IKKα protein in skin of C-IKKα and N-IKKα mice **A.** Recombinant DNA constructs employed to generate both transgenic mice lines. For C-IKKα mice generation, the nuclear localization signal (NLS) was removed from the sequence of the human IKKα cDNA employed. In the construct used for generation of the N-IKKα mice an extra NLS signal was added. WT IKKα; wild type IKKα. **B.** Western blot of total protein extracts showing IKKα expression in back skin of Control and C-and N-IKKα mice. Actin was used as a loading control. **C.** Representative example of the K5 staining in back skin section of Control mice. **D-E.** Expression of exogenous IKKα protein in back skin of 1-month-old mice. Immunostaining with the NB100-56704 anti-IKKα antibody is showed; similar results were obtained with the H00001147-M04 IKKα antibody (not shown). Note the cytoplasmic expression of the transgene in the C-IKKα mice (D). By contrast, it is located in the nuclei of cells in the N-IKKα mice (E). In both types of transgenic mice the exogenous IKKα is expressed in basal keratinocytes (bk), in the outer root sheath of hair follicles (ORS) and in cells surrounding the sebaceous glands (sb). **F.** Back skin section of Control mice. The NB100-56704 antibody used does not recognize the endogenous IKKα in immunohistochemical assays. **G.** Endogenous IKKα expression in control mice using the IKKα (sc-7182) antibody. Scale bar: (C) 70 μm; (D-G) 60 μm.

We have generated transgenic mice expressing an exogenous human IKKα protein in the cytoplasmic or nuclear compartment of keratinocytes (C-IKKα and N-IKKα mice respectively) under the control of the keratin 5 (K5) promoter (Figure 1A). The K5 derived sequences included in this construct drive transgene expression to the basal cells of the epidermis and the outer root sheath of hair follicles [36]. N- and C-IKKα mice develop normally, although the latter have a characteristic phenotype of sparse and short hair. This atypical hair was comparable to that of heterozygous IKKα^+/−^ mice obtained in a similar FVB background and to a lesser extent to that of IKKα-siRNA transgenic mice [37] and data not shown.

Western blot analysis revealed increased expression of IKKα in the skin of both C-and-N-IKKα mice (Figure 1B). Systematically, the level of IKKα transgene was higher in N-IKKα than in C-IKKα mice. Immunohistochemical staining showed that the transgenic protein was expressed in both C-and-N-IKKα mice following the K5 expression pattern (Figure 1C–1E). As it was expected, C-IKKα mice express the transgene in the cytoplasm of keratinocytes (Figure 1D), while N-IKKα mice express the exogenous IKKα in the nucleus (Figure 1E). For transgenic human IKKα analysis we have used two different antibodies that detect transgenic IKKα in immunohistochemistry (NB100-56704 and H00001147-M04 antibodies, see Materials and Methods) and they both have yielded similar results. These antibodies do not recognize endogenous IKKα as noticed by the absence of signal in the epidermis of Control (non-transgenic) mice (Figure 1F). In line with the lowest levels of transgene expression detected by western blot analysis in the C-IKKα mice, immunohistochemical assays showed a weaker staining and a lower number of cells expressing the transgene in the interfollicular epidermis of C-IKKα mice compared to that in N-IKKα mice (Figure 1D, 1E). Endogenous IKKα expression was detected both in the cytoplasm and in the nucleus of suprabasal and basal epidermal keratinocytes, as well as in hair follicle keratinocytes (Figure 1G).

### C-IKKα/TgAC mice develop higher number of skin tumors of lower latency period

**Figure 2:**
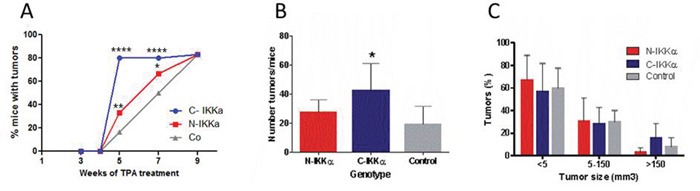
Tumors developed in C-and N-IKKα/TgAC and Control/TgAC mice **A.** Tumors emerge earlier in C-IKKα/TgAC mice than in mice of the two other genotypes. Fisher's exact test was used to determine p values (*p<0.05; **p<0.01; ***p<0.001; ****p<0.0001). **B.** Tumor multiplicity in Control/TgAC, C-IKKα/TgAC and N-IKKα/TgAC mice. Statistically significance was determined using Student's t test (*p<0.05). **C.** Representation of the percentage of tumors of the indicated size in each group of mice. Statistical analysis was determined using Bonferroni multiple comparison test. (B-C) Panels represent data obtained from the analysis of tumors harvested at 9, 14 and 19 weeks of TPA treatment.

To evaluate the effect of nuclear or cytoplasmic overexpression of IKKα on skin tumor development, we bred the three types of mice (Control, C-IKKα and N-IKKα mice) with TgAC animals. TgAC mice carry an activated Ha-*ras* transgene that triggers the classic skin tumor initiation event [38]. In this setting, double transgenic (C-IKKα/TgAC and N-IKKα/TgAC) and Control/TgAC, mice were treated with TPA (12-O-tetradecanoylphorbol-13-acetate) which promotes the expansion of *ras-*activated cells. C-IKKα/TgAc mice developed tumors earlier than the two other groups of mice; i.e. by 5-weeks of TPA treatment the percentage of mice which had developed tumors was higher (80%) in the C-IKKα/TgAC than in the N-IKKα/TgAC (33%) and Control/TgAC (16%) mice (Figure 2A). N-IKKα/TgAC mice also developed tumors a little bit earlier than Control/TgAC mice. This result indicates the lower latency period of tumor development in both types of hIKKα/TgAc mice. However, from nine weeks of TPA treatment onwards the percentage of animals that developed tumors was similar in the three groups of mice (Figure 2A). Tumors were collected at weeks 9, 14 and 19 of TPA treatment, and we found that tumor multiplicity (number of tumors/mice) was higher in C-IKKα/TgAC mice at these analyzed time points (Figure 2B). Even though we observed more tumors larger than 150 mm^3^ in C-IKKα/TgAC mice, no significant differences were found in the average tumor size in the three groups of mice (Figure 2C). Accordingly, the analysis of tumor cell proliferation, measured as BrdU incorporation, did not show significant differences between tumors of the three genotypes (data not shown). We also found no differences in apoptosis (measured as cleaved-Caspase 3 immunostaining, data not shown).

**Figure 3:**
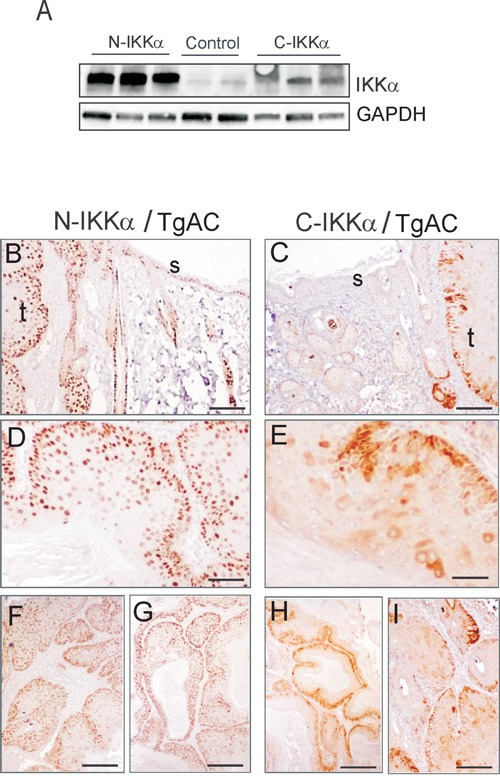
Analysis of the expression of the transgene in C-IKKα and N-IKKα tumors by biochemical and immunohistochemical approaches **A.** Western blot showing the increased expression of IKKα in transgenic mice. **B-I.** Immunohistochemistry showing the expression of the transgenic protein in N-IKKα and C-IKKa tumors. Staining with NB100-56704 antibody is shown. (B, C) Representative images showing the expression of transgenic IKKα in tumors and adjacent skin of N-IKKα/TgAC mice (B), and C-IKKα/TgAC animals (C). (D, E) Detail showing the nuclear (D) or cytoplasmic (E) localization of the transgenic IKKα in tumors. (F, G) Similar levels of expression of the transgenic IKKα in different N-IKKα tumors. By contrast variable levels of expression of the transgene are observed between different C-IKKα tumors (H, I). t: tumor; s: non-tumoral skin. Scale bar: (B, C) 100μm; (D, E) 80 μm; (F-I) 200 μm.

Western blot analysis showed that the exogenous IKKα was expressed in both N-and C-IKKα tumors, although as in the case of normal skin, the transgene was more intensely expressed in the N-IKKα tumors (Figure 3A). Immunohistochemical analysis of the transgenic IKKα protein in tumors and in the adjacent skin showed a widespread expression of the transgene in the interfollicular epidermis and hair follicles of N-IKKα/TgAC mice, similarly to the staining observed in the skin of N-IKKα mice (Figure 3B, 1E). In agreement with the high expression of the transgenic IKKα in the skin, 100% of the N-IKKα tumors analyzed (35 tumors) strongly expressed the transgenic protein at similar levels (Figure 3B, 3D, 3F, 3G). By contrast, the analysis of the skin of C-IKKα/TgAC mice showed that only some keratinocytes of the interfollicular epidermis and hair follicles expressed the transgene (Figure 3C). This result suggests that there is a negative selective pressure for survival of keratinocytes overexpressing IKKα in the cytoplasm or a tendency to silence the transgene. Surprisingly, despite the small number of keratinocytes expressing the transgene in the skin of C-IKKα/TgAC mice, almost 80% of the C-IKKα tumors tested (33/41) expressed the transgenic protein, although high heterogeneity in the levels of expression was observed (evaluated by the intensity and extent of transgenic IKKα signal) (compare Figure 3E, 3H, 3I). These results suggest that the overexpression of IKKα in the cytoplasm of keratinocytes greatly favors keratinocyte transformation and skin tumor development.

**Figure 4:**
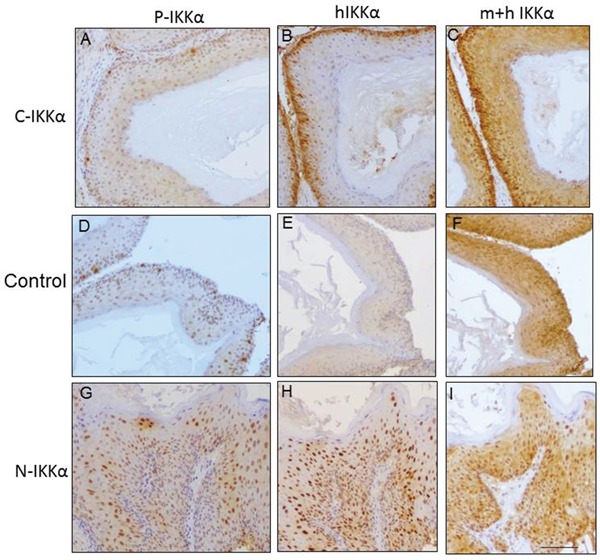
Analysis of phosphorylated P-IKKα, and human and mouse IKKα in tumors of the three groups **A, D, G.** P-IKKα expression. P-IKKα/β (Ser 180/Ser 181) antibody is used. **B, E, H.** Specific staining of human IKKα-using the NB100-56704 antibody. **C, F, I.** Staining with the sc-7182 antibody that recognizes both human and mouse IKKα. Observe that as expected, in the N-IKKα tumors the signal of this antibody is detected both in cytoplasmic and nuclear localization; by contrast, in the Control tumors the endogenous IKKα is mainly observed in the cytosolic compartment, although some nuclear staining is also observed. In the C-IKKα tumors little nuclear staining is observed. Scale bar: 70μm.

We next analyzed the presence of phosphorylated (activated) IKKα in the nucleus of tumors. A few scattered positive cells were found in the C- IKKα tumors (Figure 4A). More abundant phosphorylated IKKα was detected in Control-tumors (Figure 4D), being the N-IKKα neoplasias those presenting more frequently activated IKKα (Figure 4G). Although we used an antibody that recognized both, activated IKKα and IKKβ proteins, however, IKKβ is almost not detected in the nucleus of tumors of the three genotypes (data not shown), indicating that the staining observed in Figure 4 (A, D, G) corresponds to P-IKKα protein.

### Tumors developed in both N-and-C-IKKα/TgAC mice show increased progression than those developed in control/TgAC mice

**Figure 5:**
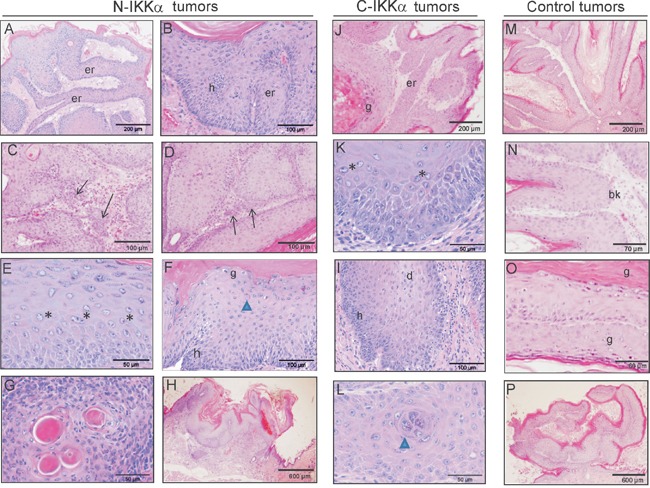
Histological pattern of tumors developed by double N-IKKα- and C-IKKα/TgAc, and Control/TgAC mice **A-H.** Hematoxilin/eosin staining of N-IKKα tumors. (A, B) Characteristic growth pattern showing networks of epidermal ridges and basal hyperplasia (B). (C, D) Foci of infiltration. (E, F) Representative image showing anisokaryosis: nuclei of very different size are observed per field. (G) Keratin pearls. (H) Example of endoexophytic pattern of tumor growth. **J-L.** Hematoxilin/eosin staining of C-IKKα tumors. (J) Representative example of epidermal ridges in the C-IKKα tumors. (K) Anisokariosis in C-IKKα tumor cells. **I.** Basal hyperplasia and dyskeratotic cells in C-IKKα tumors. (L) Attempted keratin pearl formation. **M-P.** Hematoxilin/eosin staining of Control tumors. (M) Representative pattern of growth of control tumors. (N-O) Note the absence of basal hyperplasia and the presence of stratum granulosum in Control tumors. (P) Example of exophytic pattern of tumor growth. er: epidermal ridges; h: hyperplasia; g: stratum granulosum; bk: basal keratinocytes; *: example of anisocaryosis; arrows: infiltrative focus; arrow head: formation of a keratin pearl; d: dyskeratotic cells. Tumors of 9 weeks of TPA treatment: C, D, J, N, O; Tumors of 14 weeks of TPA treatment: A, B, E, F, G, H, I, K, L, M, P.

Histopathological examination of tumors collected by 9, 14 and 19 weeks of TPA treatment was performed. Although by these times tumors were mainly papillomas, however, those arisen in both N and C-IKKα/TgAC mice exhibited a carcinomatous-like pattern of growth, forming networks of epidermal ridges growing towards the dermis (Figure 5A, 5B; Table 1). These structures were also observed in the C-IKKα tumors (Figure 5J) although they were smaller than the epidermal ridges of the N- IKKα tumors; they were not detected in the Control tumors (Figure 5M). Furthermore, in some N-IKKα/TgAC tumors infiltration foci were observed even at very early times of TPA promotion (9-weeks) (Figure 5C, 5D; Table 1). Anisokaryosis and anisocytosis (signals of tumor promotion) were found in N-IKKα tumors and, to a lesser extent, in C-IKKα tumors (Figure 5E, 5K). No significant anisokaryosis or anisocytosis was found in Control tumors (Figure 5M–5O). An important basal hyperplasia was also detected in the N-IKKα tumors (Figure 5B, 5F) and, in a minor degree, in the C-IKKα tumors, although in this case dyskeratotic cells were appreciated (Figure 5I). No relevant hyperplasia was observed in Control tumors (Figure 5N, 5O; Table 1). In the N-IKKα tumors the stratum granulosum appears discontinuous and areas exist in which the characteristic granules of this layer were not observed (indicating an altered maturation and differentiation of the keratinocytes) (Figure 5F; Table 1). Continuous stratum granulosum was observed in the C-IKKα and Control tumors (Figure 5J, 5O). Keratin pearls, characteristics of well-differentiated carcinomas, were found in N- and C-IKKα tumors (Figure 5G, 5L; Table 1). The analysis of the growth pattern of tumors showed that both, N-and C-IKKα/TgAC mice developed higher number of endoexophytic tumors than those originated in Control/TgAC mice (Table 1; 5H, 5P). This type of growth is associated to tumors prone to fast-malignization.

**Table 1:** Histological characteristics of C-IKKα, N- IKKα and Control tumors

Genotype/Tumor	C-IKKα	N-IKKα	Control	P value C-IKKα/Control	P value N-IKKα/Control
Epidermal ridges	10/23	22/25	0/23	0,0006	< 0,0001
Infiltration foci	0/23	5/25	1/23	ns	ns
Basal hyperplasia	6/23	10/25	2/23	ns	0,019
Stratum granulosum discontinuous	1/23	7/25	1/23	ns	0,049
Keratin pearls	5/23	7/25	0/23	0,049	0,01
Endoexophytic growth	30/75	40/68	17/62	ns	0.0004

Table shows the number of tumors analyzed and the number of tumors positive for each characteristic. Statistical analysis is showed. Fisher's exact test was used to determine p values.

**Figure 6:**
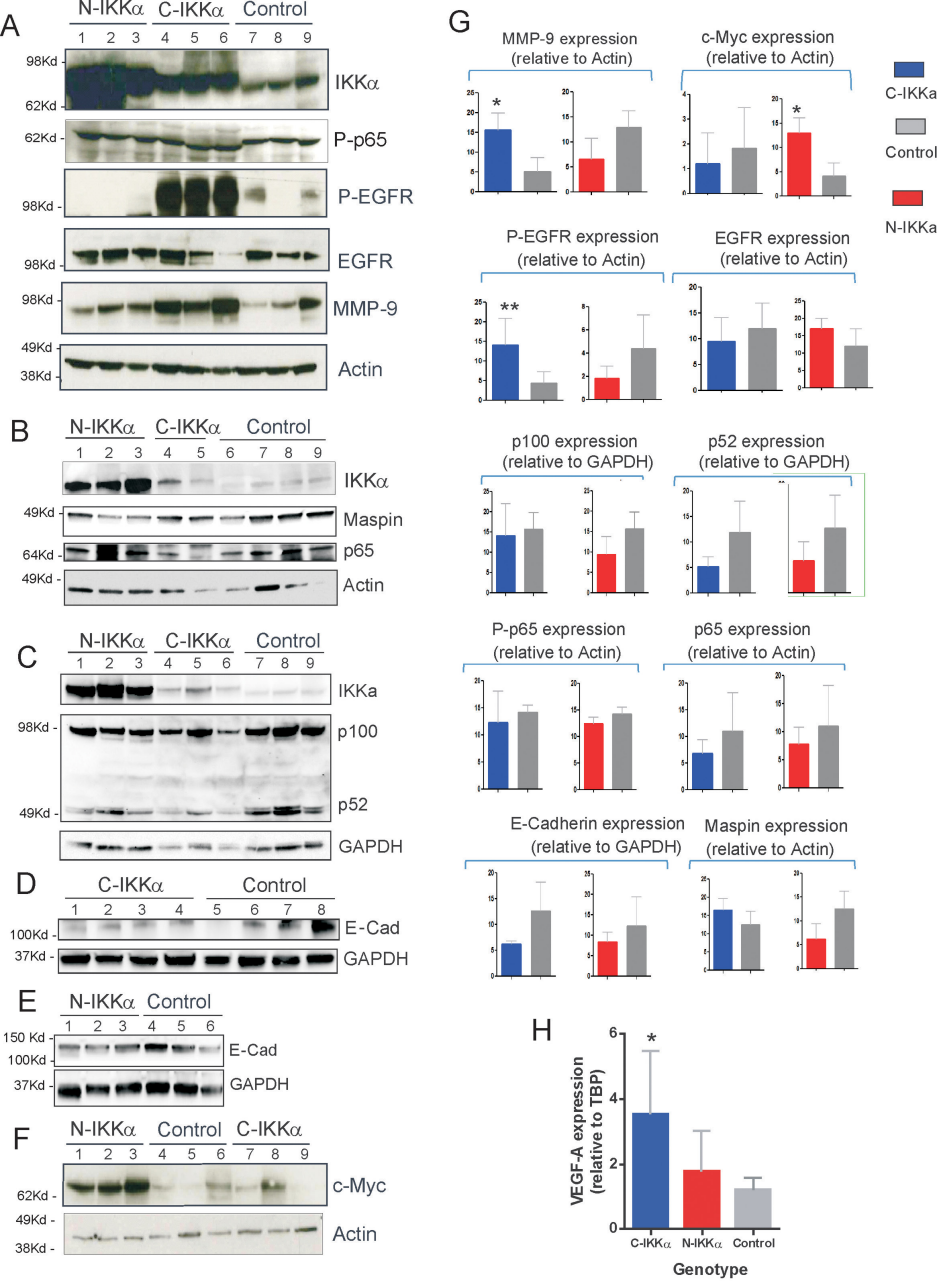
Biochemical characterization of Control, C-IKKα and N-IKKα tumors **A-F.** Representative Western blots analysis of IKKα, P-p65, p65, EGFR, P-EGFR, p100/52, Maspin, c-Myc, E-cadherin and MMP-9 expression in Control, C-IKKα and N-IKKα tumors. Actin and GAPDH were used as loading controls. Western blot of protein extracts from 5 to 8 tumors derived from to 4 to 6 different mice of each genotype were performed. The identification of each tumor and mouse corresponding to every lane is provided in Supp. data **G.** Bands of the different immunoblots were quantified by Quantity One software and Image Lab software and normalized with respect to Actin or GAPDH expression. P values were determined by Student's *t*-test and p values <0.05 (*) were considered significant; **p<0.01. **H.** Determination of VEGF-A mRNA relative levels in skin of Control, C-IKKα and N-IKKα transgenic mice by qRT-PCR analyses (*P*<0,05).

**Figure 7:**
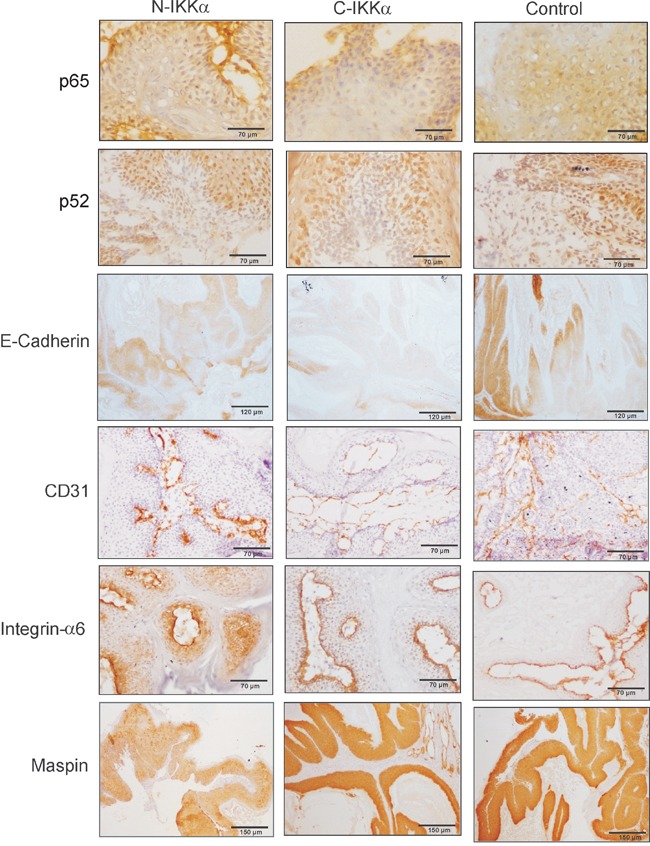
Analysis of tumor markers in Control, C-IKKα and N-IKKα tumors Downregulation of E-cadherin expression in N-IKKα- and C-IKKα tumors. The CD31 staining (marker of endothelial cells) shows the presence of dilated and leaky blood vessels in the C-IKKα tumors, while those of Control and N-IKKα tumors are narrow and mature. Strong and delocalized suprabasal integrin-a6 staining is detected in N-IKKα tumors, while Control and C-IKKα tumors show Integrin-α6 basal expression. Reduced staining of Maspin in N-IKKα tumors. No difference in p52 and p65 expression was notice between tumors of the three genotypes.

Altogether these histological results suggest that tumors developed in both N-and C-IKKα/TgAC mice have more malignant features than those arisen in Control/TgAC mice. In addition, among them, the N-IKKα tumors seem more aggressive than the C-IKKα ones. To test whether the activation of the classical NF-kB pathway causes the increased malignancy of the N-and-C-IKKα tumors, activation of p65 and total p65 levels were assessed. No differences between the three types of tumors were found (Figure 6A, 6B, 6G; Supp Figure 1A, B). As IKKα is a central protein of the alternative NF-kB pathway, the balance p100/p52 was also analyzed and no evident differences were found between tumors of the three groups (Figure 6C, 6G). Moreover, immunostaining with p52 and p65 antibodies did not reveal a significant difference in the level of expression or subcellular localization of these proteins among tumors of the three genotypes (Figure 7).

### Increased EGFR activation and enhanced VEGF-A and MMP-9 expression in C-IKKα tumors

Other markers of tumor progression were analyzed, such as the expression of E-cadherin, being those tumors expressing low levels of E-Cadherin considered of worse prognosis. The immunohistochemical staining showed a faint signal for E-Cadherin in tumors originated in both types of IKKα/TgAC mice, mainly in the C-IKKα ones (Figure 7). Western blot analysis also indicated a decrease in the expression of E-Cadherin in both types of hIKKα tumors (Figure 6D, 6E, 6G; Supp. Figure 1C). A robust tumor angiogenesis is another indicator of tumor malignancy. The analysis of CD31 and Sma immunostaining showed that C-IKKα tumors exhibited a network of large and lacunar, dilated blood vessels; by contrast, Control and N-IKKα tumors showed a blood vessels pattern characterized predominantly by narrow and small capillaries, although sporadically vessels of intermediate lumen diameter were observed in N-IKKα tumors (Figure 7). In addition, the staining of blood vessels with these antibodies showed a weak and discontinuous signal in C-IKKα tumors indicating the immature and leaky nature of their blood vessels. On the contrary, the vessels of the N-IKKα and Control tumors were more strongly stained, especially those in N-IKKα tumors (Figure 7, and data not shown). One of the most important pro-angiogenic factors is VEGF-A (vascular endothelial growth factor-A). As it has been described that IKKα represses VEGF-A expression in the skin [33], we analyzed the levels of VEGF-A expression in the skin of the three groups of mice. Whereas similar levels of VEGF-A mRNA in both Control- and N-IKKα mice were found, we observed a significant induction of VEGF-A expression in the skin of C-IKKα mice (Figure 6H). This result contrasts with a report from another group [33], but it is in agreement with the expanded network of blood vessels detected in the C-IKKα tumors. Searching for a possible cause of this increase, we analyzed the expression of two positive regulators of VEGF-A expression in tumors, i.e. EGFR (Epidermal Growth Factor Receptor) [39–41], and MMP-9 (metalloproteinase 9) [42]. We found no significant differences in the levels of EGFR expression between tumors developed by mice of the three genotypes; however, EGFR activation (P-EGFR) was augmented in the C-IKKα tumors (Figure 6A, 6G; Supp. Figure 1D). Enhanced MMP-9 expression was also observed in tumors arisen in C-IKKα/TgAC mice (Figure 6A, 6G; Supp. Figure 1G), indicating that upregulation of these two factors could cause the observed increase in VEGF-A.

Interestingly, increased levels of VEGF-A expression and augmented angiogenesis was also found in tumors developed in mice expressing reduced levels of IKKα [29]. Furthermore, induction of EGFR appears to be responsible for the skin tumor development in IKKα^F/F^/K15.Cre mice lacking IKKα in keratinocytes [28]. Upregulation of MMP-9 gene has also been found in IKKα^F/F^/K5.Cre keratinocytes, and has been associated to keratinocyte transformation [28]. Thus, our results suggest that C-IKKα mice overexpressing IKKα in the cytoplasm of keratinocytes, and transgenic mice expressing reduced levels of IKKα promote the development and progression of NMSC by similar mechanisms.

### Induction of c-Myc, downregulation of Maspin levels and delocalized expression of Integrin-α6 in N-IKKα tumors

In a previous work we described that WT IKKα overexpression in both nucleus and cytoplasm of keratinocytes of K5-IKKα transgenic mice increased the malignant potential of skin tumors [30]. We identified the downregulation of the tumor suppressor and repressor of metastasis Maspin, and the induction of delocalized suprabasal expression of Integrin-α6, as the mechanisms through which IKKα exerted its protumoral function. Now we have analyzed the expression of these proteins in tumors of the three groups of mice. While in benign NMSC Integrin-α6 is expressed in keratinocytes of the basal layer; however, in malignant skin tumors it is also expressed in suprabasal layers [43]. We observed that Control and C-IKKα tumors had basal staining of Integrin-α6 (Figure 7) while tumors from N-IKKα/TgAC mice exhibited basal as well as delocalized suprabasal expression of Integrin-α6 (Figure 7). The suppressor of metastasis Maspin was expressed at lower levels in the N-IKKα tumors compared with Control- and C-IKKα tumors (Figure 7). A decrease in Maspin levels was also found by Western blot analysis (Figure 6B, 6G; Sup Figure 1F, G). Therefore these data support the more aggressive feature of the tumors developed in N-IKKα/TgAC mice and that tumors arisen in N-IKKα/TgAC mice share molecular characteristics with those developed in the K5-IKKα/TgAC mice [30].

One important molecule for keratinocyte transformation and tumor progression that is regulated by IKKα is the proto-oncogene c-Myc [44–46]. Analysis by Western blot has shown that c-Myc expression is induced in the N-IKKα tumors (Figure 6F, 6G; Sup Figure 1F). This data reinforces the above results suggesting the increased malignancy of tumors developed by the N- IKKα/TgAC mice, and is in agreement with data showing the gain of c-Myc copy number gene found in moderately to poorly differentiated SCCs when compared with well-differentiated SCCs [47]. Therefore, the immunohistochemical and biochemical studies confirm the histopathological results, indicating that N-IKKα/TgAC mice develop tumors of increased aggressiveness than Control/TgAC mice.

**Figure 8:**
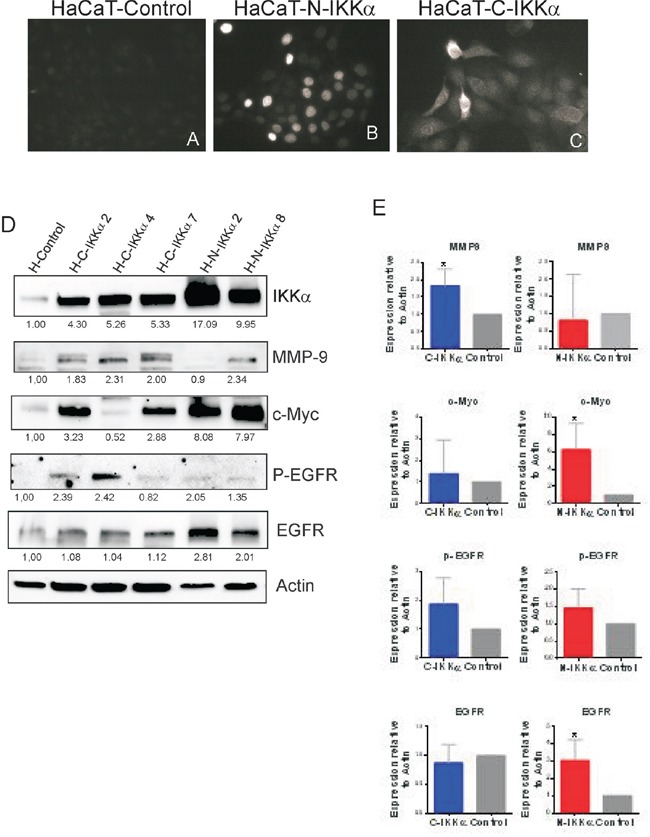
Characterization of the HaCaT-N-IKKα and HaCaT-C-IKKα cells **A-C.** Immunofluorescence with a Flag specific antibody showing the expression of the transgene in the nucleus of the HaCaT-N-IKKα cells (B) and in the cytoplasm of the HaCaT-C-IKKα cells (C). **D.** Representative western blot analyisis showing increased levels of IKKα in different pools of transfected HaCaT clones. Observe the increased MMP-9 and EGFR activation in the HaCaT-C-IKKα cells and the enhanced expression of c-Myc in the HaCaT-N-IKKα cells. **E.** Graphic representation of the densitometric analysis of western blots correponding to 6 pooled clones of HaCaT-C-IKKα cells, 3 pooled clones of HaCaT-N-IKKα cells and 3 pooled clones of HaCaT-Control cells. Student's t test was used for statistical analysis. (*p<0.05; ****p<0.0001).

To confirm that the differences in the c-Myc, P-EGFR and MMP-9 expression found in N-and-C-IKKα tumors were due to the expression of the N-IKKα and C-IKKα transgenes, we transfected the HaCaT cell line of human keratinocytes with the C-IKKα and N-IKKα constructs under the control of the β-Actin promoter. Pooled stable transfectans clones from 15-30 different colonies were used to minimize any potential effect of clonal selection. A total of 6 pooled HaCaT-C-IKKα clones, 3 pooled HaCaT-N-IKKα clones and 3 pooled HaCaT-Control clones were analyzed. The correct expression of the transgene in the nucleus of HaCaT-N-IKKα and in the cytoplasm of the HaCaT-C-IKKα was determined by immunofluorescence analysis (Figure 8A–8C). Western blot analysis confirmed the increased expression of IKKα in the transfected cells (Figure 8D). Similarly to N-IKKα tumors obtained in transgenic mice, we observed that HaCaT-N-IKKα cells expressed increased levels of c-Myc; additionally we detected augmented EGFR activation and enhanced expression of MMP-9 in HaCaT-C-IKKα cells (Figure 8D, 8E; Supp. Figure 2), similarly to the results found in the C-IKKα tumors. Therefore, these results suggest that the increase in the expression of c-Myc in the N-IKKα tumors and in the HaCaT-N-IKKα keratinocytes is likely due to augmented IKKα expression of nuclear localization in keratinocytes. In addition they also suggest that the increase in MMP-9 levels and P-EGFR activation found in the C-IKKα tumors and in the HaCaT-C-IKKα cells is mainly originated by the cytoplasmic localization of IKKα in keratinocytes.

In summary, our results show that tumors developed in both C-IKKα and N-IKKα/TgAc mice overexpressing IKKα in the cytoplasm or the nucleus of keratinocytes respectively are more prone to tumor development and have more aggressive features than those developed in Control/TgAC mice. Indeed, at the time of submitting this manuscript, we have started to observe spontaneous development of skin tumors in aged C-and-N-IKKα mice, strongly reinforcing the role of IKKα as a tumor promoter of NMSC in either, cytoplasmic or nuclear localization (work in progress). Importantly, our molecular studies, *in vitro* and *in vivo*, suggest that IKKα exerts very different and defined functions in each subcellular compartment of keratinocytes.

## DISCUSSION

Our models of transgenic mice expressing an exogenous IKKα protein in the cytoplasm or in the nucleus of keratinocytes provide an excellent model for discerning the function that IKKα develops in NMSC. Our results show that regardless of its subcellular localization, IKKα plays a protumoral role in skin cancer development and progression, although the mechanisms through which IKKα promotes NMSC are different depending on its nuclear or cytoplasm localization.

Interestingly, we have found that in spite of the low number of keratinocytes that express the transgenic IKKα protein, C-IKKα/TgAC mice developed larger number of skin tumors with lower latency than Control/TgAC mice. This result suggests a very high predisposition for malignant transformation of keratinocytes expressing cytoplasmic IKKα. Additionally, this data proposes that the low expression of transgene expression in the epidermis of C-IKKα mice may be the result of a negative selection against keratinocytes expressing the transgenic IKKα, favoring the expansion of those in which the transgene is silenced. We have found that the increased levels of VEGF-A, along with the associated increased angiogenesis, the decreased expression of E-Cadherin and the enhanced expression of MMP-9 in the C-IKKα tumors are the likely mechanisms that may lead to the further progression found in C-IKKα tumors. In this respect, the induction of MMP-9 expression through a PI3K/Akt/IKKα pathway has been previously described [48]. In addition, an association between reduced expression of E-Cadherin and increased MMP-9 levels has been reported in mouse SCCs with invasive phenotype [49]. These features are also common in human NMSC, where increased MMP-9 expression has been observed in SCCs with respect to benign lesions in BCCs and actinic keratosis [50].

Another feature that distinguishes C-IKKα tumors from Control and N-IKKα tumors is the increased activation of EGFR. IKKα has been shown to be integrated into the EGFR/Ras/Erk pathway during mitosis and differentiation as well as in skin cancer development [51]. EGFR activation has been associated with tumor progression in different types of cancer in humans and mice, including NMSC, and usually correlates with a worse prognosis [39]. Moreover, increased MMP-9 and EGFR activation could be enhancing tumor angiogenesis in the C-IKKα tumors as both molecules are involved in processes required for tumor invasion of tissues and metastasis, such as angiogenesis [52, 53]; they are also positive regulators of VEGF-A expression [41, 42], that predisposes murine epidermis to NMSC development [54, 55] and favor the growth of large and lacunar blood vessels (a prominent feature of skin tumor progression) [56].

It is striking that the features that distinguish skin tumors developed by C-IKKα/TgAC mice, i.e., MMP-9 and VEGF-A upregulation and increased EGFR activation are the same that characterize the skin tumors arisen in mice lacking IKKα or expressing diminished levels of this protein [28, 29]. It is also remarkable that our C-IKKα mice express increased levels of VEGF-A, since it has been shown that IKKα binds in the nucleus of keratinocytes to the distal VEGF-A promoter repressing its expression [33]. These results suggest that overexpression of IKKα in the cytoplasm of keratinocytes may impair certain nuclear IKKα functions (such as VEGF-A regulation), resulting in a similar behaviour of C-IKKα tumors and those developed in IKKα null mice (that consequently are deficient in the nuclear IKKα function). This possibility will be analyzed in detail in future work.

Our results show that tumors developed in N-IKKα/TgAC mice also present a more aggressive phenotype than those of Control/TgAC mice. As a possible mechanism for this increased tumor progression, we have found the induction of c-Myc expression. In support of this hypothesis, a mechanistic connection between IKKα and c-Myc has been found in breast cancer, where IKKα increases c-Myc protein levels by prolonging protein stability, and this consequently promotes the tumorigenic and invasive activity of breast cancer cells [57]. In the case of NMSC, increased c-Myc expression is found in SCC of poor prognosis [47]. In addition, the expression of this proto-oncogene in epidermis is a risk factor for tumor development, i.e., K5-cMyc transgenic mice develop spontaneous tumors in skin and oral cavity [44, 58]. c-Myc amplification or deregulated expression can also play a causal role in the genesis and tumorigenic promotion of diverse human tumors, including cutaneous SCC, lung and breast carcinomas [47, 59–62]. Actually, c-Myc amplification has been found in 50% of tumors from transplants recipients who develop skin SCC [63], being the incidence of cutaneous SCC development highly augmented in these patients [64].

Another event that may account for the increased tumor progression of N-IKKα tumors is the decreased expression of the tumor suppressor Maspin. Maspin acts as an inhibitor of metastasis in prostate, liver and breast cancers, in which it has been proven that nuclear IKKα, by directly binding to the Maspin promoter, represses its transcription, thereby encouraging metastasis [24, 26, 65]. Previous findings of our group showed that Maspin also has a tumor suppressor role in NMSC development and progression, and furthermore we found that the increased malignancy of tumors developed in K5-IKKα/TgAC mice could be due to the diminished expression of Maspin in skin [30, 66]. Hence, the results that we here show confirm our previous data and extend our findings, as we have specified the role of nuclear IKKα as a regulator of Maspin expression in skin tumors developed by N-IKKα/TgAC mice.

Recently, it has been published that the nuclear localization of IKKα is a hallmark of aggressive human cutaneous SCC with high risk to metastasize [67]. These authors have found that nuclear IKKα is coupled with the metastatic capacity of cutaneous SCC, likely through Maspin attenuation. These results agree with the increased aggressiveness of skin tumors overexpressing nuclear IKKα expression in comparison to Control tumors described here, and confirm the importance of reduced levels of Maspin for the enhanced aggressiveness of these tumors. In addition, we have found other mechanisms that are likely contributing to the high malignancy of skin tumors with increased nuclear IKKα, such as induction of c-Myc expression and deregulation of Integrin-α6 (a common feature found in squamous tumors at high risk of malignant progression [43]). Therefore, our results show that N-IKKα tumors recapitulate some of the features that make human and mouse skin tumors more aggressive and with high risk to metastasize.

In summary, our models of transgenic mice show that regardless of its subcellular localization in keratinocytes, IKKα plays a protumoral role in skin cancer development and progression, although by different mechanisms. We have also found interesting similarities between the features of tumors developed in C-IKKα mice and those of tumors arising in IKK^−/−^ mice. And importantly, we have found that N-IKKα skin tumors mimic the characteristics associated to aggressive human skin tumors with high risk to metastasize such as predominance of nuclear IKKα expression and attenuation of Maspin expression, besides the induction of c-Myc and Integrin-α6 expression. Our results may help in understanding the progression of human NMSC and also offer new targets for intervention in such common cancer in humans.

## MATERIALS AND METHODS

### Generation of transgenic mice

The human IKKα cDNA sequence was amplified from human keratinocyte RNA and cloned in pCRII-TOPO using Topo-Cloning (ThermoFisher, MA, USA) using specific primers that also included restriction sites (HindIII in 5′ and NotI in 3′). For N-IKKα cloning, the primer also included an NLS (nuclear localization signal) (atggatcccaagaagaagaggaaggtg) in 5′. For C-IKKα, the internal NLS site was removed by directed mutagenesis using QuikChange II (Stratagene). All constructs were checked by sequencing. N-IKKα and C-IKKα constructs were then subcloned in the pK5 vector containing 5.2 Kb of the bovine K5 promoter and a rabbit β-globin intron (Figure 1A) [36].

C-IKKα and N-IKKα mice were generated in FVB/N and B6D2F2 hybrid background respectively. N-IKKα mice were then crossed with FVB/N mice and used in the 6^th^ generation onwards. Mice were genotyped by PCR analysis of tail genomic DNA using primers specific for the rabbit β-globin intron.

### Carcinogenesis assays

Female hemizygous v-Ha-*ras* transgenic Tg.AC mice (Taconic Farm Inc. USA) were mated with C- and N-IKKα, and Control males. Double transgenic C-and N-IKKα/Tg.AC and Control/Tg.AC 9-week-old mice (6 animals per group) were shaved and topically treated twice weekly with 5 μg of TPA (Sigma) in 200 μl of acetone for 9 weeks according to standard protocols [30]. Tumors were measured with an external caliper, and volume was calculated as (4/3) π (width/2)^2^ (length/2).

### Cell culture and transfection assays

Human HaCaT keratinocytes were growth in DMEM and 10%FCS. Cells were transfected using the calcium phosphate method. N-IKKα and C-IKKα constructs (containing a Flag-tag) were then subcloned in a vector containing the β-Actin promoter [31, 68, 69]. The corresponding empty vector was used as control. Resistant colonies were selected using G418 (0.5 mg/ml). As a result of different transfection assays distinct clones of HaCaT cells were obtained expressing the N-IKKα or C-IKKα transgenes, designated HaCaT- N-IKKα and HaCaT-C-IKKα. Each clone was derived from a pool of 15-30 different colonies. HaCaT colonies transfected with the empty vector were selected, pooled, and used as control (HaCaT-Control cells).

### Histology and immunohistochemistry

Skin and tumors were fixed in 10% buffered formalin and embedded in paraffin. Sections were stained with H&E and histopathological evaluation was performed by two experimented observers: MJFA, specialized in human pathology and RAGF, a veterinarian expert in animal pathology.

Immunostaining was performed using antibodies against IKKα (NB100-56704) IKKβ (Novus Biologicals, Cambridge UK); IKKα (H00001147-M04) (Abnova, Taiwan); IKKα (sc-7182), P-IKKα/β (Ser 180/Ser 181)-R (sc-23470-R), Maspin, p65 (Santa Cruz Biotechnology, Inc. Heidelberg, Germany); CD31, E- Cadherin, Integrin-α6 (BD Bioscience, NJ, USA); p52 (Abcam, Cambridge, UK). Sections were incubated with a biotinylated secondary antibody, and then with streptavidin conjugated to horseradish peroxidase (DAKO A/S, Glostrp, Denmark). Antibody localization was determined using 3,3-diaminobenzidine (DAB) (Vector Laboratories; Burlingame, CA, USA).

A pressure cooker with DAKO target retrieval solution ph9.0 (DAKO) was employed for Maspin, mouse IKKα, human IKKα, P-IKKα/β, IKKβ and E- Cadherin detection. Staining with p52, p65, Integrin-α6 and CD31 antibodies was performed in cryosections of tumors.

### Immunofluorescence

Indirect immunofluorescence was used to detect the transgene in HaCaT cell cultures. The Flag antibody was used (F3040; SIGMA, Missouri, USA). Alexa Fluor-594 goat antimouse IgG(H + L) was used as fluorochrome.

### Ethics statement

All animal experimental procedures were performed according to European and Spanish laws and regulations (2007/526/CE) and approved by our institution's ethics committee.

### Western blot analysis

Protein extracts were obtained from pieces of tumors or from HaCaT cells. Total protein extracts (30 μg) were subjected to SDS/PAGE. The separated proteins were transferred to nitrocellulose membranes (Amersham, Arlington Heights, IL; BioRad, France) and probed with antibodies against IKKα (NB100-56704 Novus Biologicals); c-Myc (Biolegend, CA, USA); Maspin, Actin, EGFR, P-EGFR (Tyr1176), p65, GAPDH (Santa Cruz Biotechnology, Inc. Europe); α-Tubulin (Sigma-Aldrich, MO, USA); E-Cadherin (BD Bioscience, NJ, USA). p100/p52 (Cell Signaling Technology, USA) and MMP-9 (Merck Millipore, Darmstadt, Germany). In all cases samples were subjected to luminography with the Supersignal West Pico Chemiluminescent Substrate (Pierce Biotechnology, Inc., Illinois, USA).

### RNA isolation and real-time PCR

Total RNA was isolated using miRNeasy Mini Kit (Qiagen, Hilden, Germany) according to the manufacturer's instructions and residual DNA was eliminated using Rnase-Free Dnase Set (Qiagen). Reverse transcription was performed using the Omniscript RT Kit (Qiagen) and oligo dT primer, using 1 μg of total RNA. Quantitative PCR was performed in a 7500 Fast Real Time PCR System (Applied Biosystems, Foster City, CA, USA) using Go Taq PCR master mix (Promega) and 1 μl of cDNA as a template. Melting curves were performed to verify specificity and absence of primer dimerization. Reaction efficiency was calculated for each primer combination, and TBP gene was used as reference gene for normalization. The F-and R-sequences of the specific oligonucleotides for VEGF-A were 5′-CAGGCTGCTGTAACGATGAA-3′and 5′-CTCCTATGTGCTGGCTTTGG-3′ and for TBP were 5′-AGTGAAGAACAGTCCAGACTG-3′ and 5′-CCAGGAAATAACTCTGGCTCAT-3′.

## SUPPLEMENTARY MATERIAL FIGURES


