# Correction: Deciphering the role of nuclear and cytoplasmic IKKα in skin cancer

**DOI:** 10.18632/oncotarget.20001

**Published:** 2017-08-07

**Authors:** Josefa P. Alameda, Miriam Gaspar, Ángel Ramírez, Manuel Navarro, Angustias Page, Cristian Suárez-Cabrera, M. Guadalupe Fernández, Jose R. Mérida, Jesús M. Paramio, Rosa A. García-Fernández, M. Jesús Fernández-Aceñero, M. Llanos Casanova

Copyright: Alameda et al. This is an open-access article distributed under the terms of the Creative Commons Attribution License 3.0 (CC BY 3.0), which permits unrestricted use, distribution, and reproduction in any medium, provided the original author and source are credited.

**Present:** The grant support information is incomplete.

**Correct:** The complete grant support is as follows.

Original article: Oncotarget. 2016; 7:29531-29547. https://doi.org/10.18632/oncotarget.8792

FUNDING

This research was supported partially by funds from Fondo Europeo de Desarrollo Regional (FEDER) and by grants from the Ministry of Economy and Competitiveness of Spain (PI13/02580 and PI14/01403, from the Instituto de Salud Carlos III to M.L. Casanova and A. Ramirez respectively) and by grants 1.010.511 (Fundacion Banco de Santander-Universidad Alfonso X el Sabio) to M.J Fernandez-Acenero; Comunidad Autonoma de Madrid grant S2010/BMD-2470 (Oncocycle Program) and CIEM13-4E-1944 to JMP; AES grants ISCIII-RETIC RD06/0020/0029 and RD12/0036/0009 to JMP.

