# Supplementary material for: Deciphering the role of nuclear and cytoplasmic IKKα in skin cancer
Source: Oncotarget. 2016 Apr 18;7(20):29531–47. doi: 10.18632/oncotarget.8792 (PMC5045415; doi:10.18632/oncotarget.8792)
Supplement: Supplementary file 1 [file oncotarget-07-29531-s001.pdf]

Deciphering the role of nuclear and cytoplasmic IKKα in skin cancer

SUPPLEMENTARY MATERIAL

Supplementary data

Identification of each tumor and mouse corresponding to every lane. Information about tumors and mice analyzed in the Figure 6. t=tumor; m=mouse.

(A) Lanes 1-3, N-IKKα mice; lanes 4-6, Control mice; lanes 7-9, C-IKKα mice. t= tumor; m= mouse. Lane 1, t 7, m A658; lane 2, t 10, m A658; lane 3, t 11, m A658; lane 4, t 1, m A633; lane 5, t 4, m A633; lane 6, t 3, m A654; lane 7, t 1, m A645; lane 8, t 9, m A688; lane 9, t 10, m A694.

(B) Lanes 1-3, N-IKKα mice; lanes 4-6, C-IKKa mice; lanes 7-9, Control mice. t= tumor; m= mouse. Lane 1, t 5, m A629; lane 2, t 14, m A653; lane 3, t 13, m A659; lane 4, t 2, m A643; lane 5, t 4, mA643; lane 6, t 8, mA632; lane 7, t 6, m A693; lane 8, t 10, m A693; lane 9, t 24, m A693.

(C) Lanes 1-3, N-IKKα mice; lanes 4-6, C-IKKα mice; lanes 7-9, Control mice. t= tumor; m= mouse. Lane 1, t 5+t6, m A653; lane 2, t 10, m A658; lane 3, t 11, m A658; lane 4, t 4, m A643; lane 5, t 9, m A688; lane 6, t 1, m A694; lane 7, t 1, m A633; lane 8, t 4, m A633; lane 9, t 10, m A654.

(D) Lanes 1-5, C-IKKα mice; lanes 6-8, Control mice. t= tumor; m= mouse. Lane 1, t 6492, m A652; lane 2, t 6493, m A652; lane 3, t4, m A652; lane 4, t 1, m A694; lane 5, t 1, m A633; lane 6, t 4, m A633; lane 7, t 10, m A633; lane 8, t 1, m A654.

(E) Lanes 1-3, N-IKKα mice; lanes 4-6, Control mice. t= tumor; m= mouse. Lane 1, t 11, m A658; lane 2, t 5+t 6, m A653; lane 3, t 15+t 17, m A653; lane 4, t 7, m A633; lane 5, t 6506, m A648; lane 6, t 31, m A693.

(F) Lanes 1-3, N-IKKa mice; lanes 4-6, Control mice; lanes 7-9, C-IKKα mice. t= tumor; m= mouse. Lane 1, t 7, m A658; lane 2, t 10, m A658; lane 3, t 11, m A658; lane 4, t 1, m A633; lane 5, t 4, m A633; lane 6, t 3, m A654; lane 7, t 1, m A645; lane 8, t 9, m A688; lane 9, t 10, m A694.

SUPPLEMENTARY FIGURES

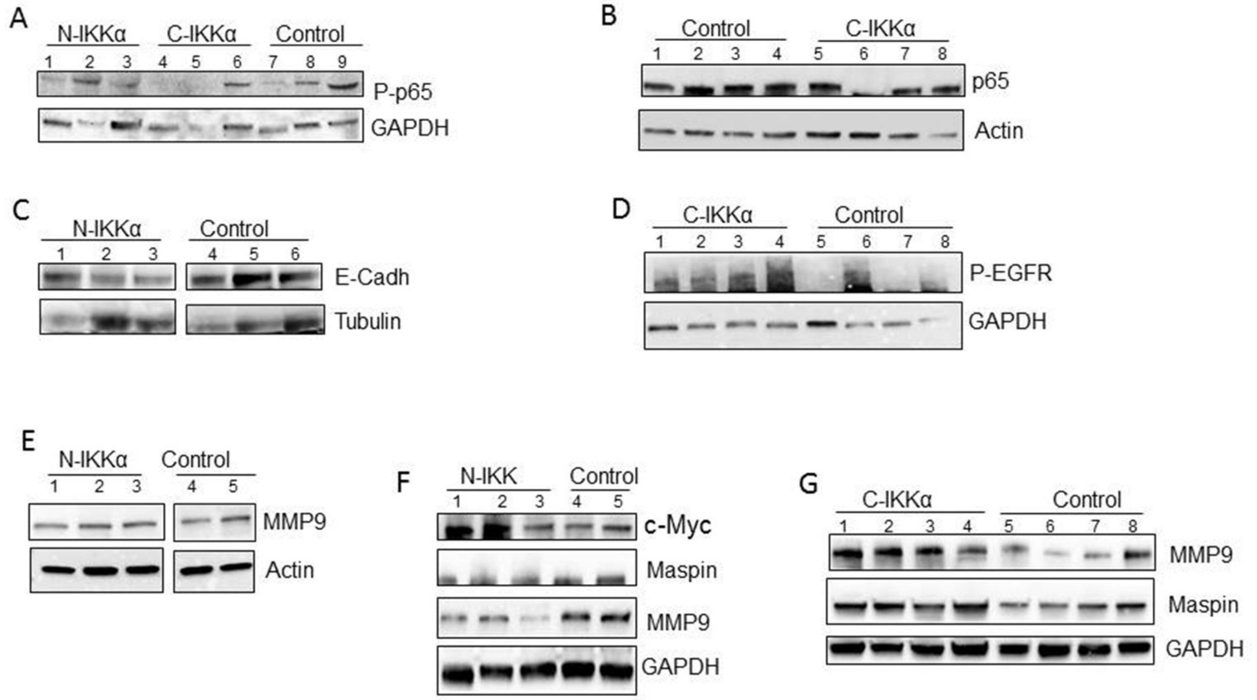

Supplementary Figure S1: Western blot analysis of proteins in Control, C-IKKα and N-IKKα tumors. Actin, Tubulin or GAPDH were used as loading controls. Tumor and mouse corresponding to every lane (t, tumor; m=mouse): **A.** Lanes 1-3, N-IKKα mice; lanes 4-6, C-IKKα mice; 7-9, Control tumors. t= tumor; m= mouse. Lane 1, t 11, m A658; lane 2, t 5+t 6, m A653; lane 3, t 5, m A629; lane 4, t 4, m A643; lane 5, t 9, A688; lane 6, t 6492, m A652; lane 7, t 4, m A633; lane 8, t 1, m A654; lane 9, t 31, m A693. **B.** Lanes 1-4, Control mice; lanes 5-8, C-IKKα mice. t= tumor; m= mouse. Lane 1, t 6520, m A627; lane 2, t 6499, m A648; lane 3, t 1, m A648; lane 4, t2, m A648; lane 5, t 6489, m A652; lane 6, t 6490, m A652; lane 7, t 1, m A652; lane 8, t 2, m A652. **C.** Lanes 1-3, N-IKKα mice; lanes 4-6, Control mice. t= tumor; m= mouse. Lane 1, t 11, m A658; lane 2, t 5+t 6, m A653; lane 3, t 5, m A629; lane 4, t 4, m A633; lane 5, t 1, m A654; lane 6, t 31, m A693. **D.** Lanes 1-4, C-IKKα mice; lanes 5-8, Control mice. t= tumor; m= mouse. Lane 1, t 4, m A643; lane 2, t 1, m A694; lane 3, t 9, m A688; lane 4, t 1, m A694; lane 5, t 1, m A654; lane 6, t 10, m A633; lane 7, t 4, m A633; lane 8, t 21, m A645. **E.** Lanes 1-3, N-IKKα mice; lanes 4,5, Control mice. t= tumor; m= mouse. Lane 1, t 5, m A629; lane 2, t 14, m A653; lane 3, t 13, m A659; lane 4, t 6, m A693; lane 5, t 10, m A693. **F.** Lanes 1-3, N-IKKα mice; lanes 4-5, Control mice. t= tumor; m= mouse. Lane 1, t 11, m A658; lane 2, t 5+t 6, m A653; lane 3, t 15+t 17, m A653; lane 4, t 7, m A633; lane 5, t 6506, m A648. **G.** Lanes 1-4, C-IKKα mice; lanes 5-8, Control mice. t= tumor; m= mouse. Lane 1, t 6492, m A652; lane 2, t 6493, m A652; lane 3, t4, m A652; lane 4, t 1, m A694; lane 5, t 1, m A633; lane 6, t 4, m A633; lane 7, t 10, m A633; lane 8, t 1, m A654.

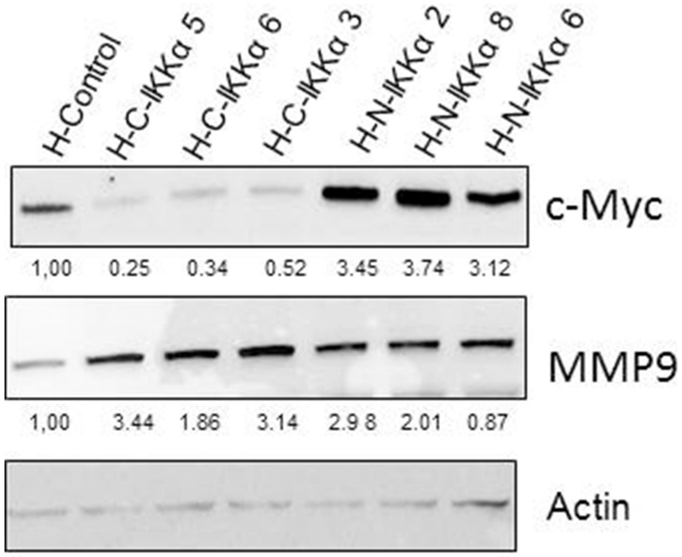

Supplementary Figure S2: Western blot analysis of additional HaCaT-C-IKKα and HaCaT-N-IKKα pooled clones. Actin was used as loading control.
